# Study of the vertical transport in *p*-doped superlattices based on group III-V semiconductors

**DOI:** 10.1186/1556-276X-6-175

**Published:** 2011-02-25

**Authors:** Osmar FP dos Santos, Sara CP Rodrigues, Guilherme M Sipahi, Luísa MR Scolfaro, Eronides F da Silva Jr

**Affiliations:** 1 Departamento de Física, Universidade Federal Rural de Pernambuco, R. Dom Manoel de Medeiros s/n, 52171-900 Recife, PE, Brazil; 2 Instituto de Física de São Carlos, USP, CP 369, 13560-970, São Carlos, SP, Brazil; 3 Department of Physics, Texas State University, 78666 San Marcos, TX, USA; 4 Departamento de Física, Universidade Federal de Pernambuco, Cidade Universitária, 50670-901, Recife, PE, Brazil

## Abstract

The electrical conductivity σ has been calculated for *p*-doped GaAs/Al_0.3_Ga_0.7_As and cubic GaN/Al_0.3_Ga_0.7_N thin superlattices (SLs). The calculations are done within a self-consistent approach to the $\vec{k}\cdot \vec{p}$ theory by means of a full six-band Luttinger-Kohn Hamiltonian, together with the Poisson equation in a plane wave representation, including exchange correlation effects within the local density approximation. It was also assumed that transport in the SL occurs through extended minibands states for each carrier, and the conductivity is calculated at zero temperature and in low-field ohmic limits by the quasi-chemical Boltzmann kinetic equation. It was shown that the particular minibands structure of the *p*-doped SLs leads to a plateau-like behavior in the conductivity as a function of the donor concentration and/or the Fermi level energy. In addition, it is shown that the Coulomb and exchange-correlation effects play an important role in these systems, since they determine the bending potential.

## Introduction

The transport phenomena in semiconductors in the direction perpendicular to the layers, also known as vertical transport, have been investigated in recent years from both experimental and theoretical points of view because of their increased application in the development of electro-optical devices, lasers, and photodetectors [1-3]. The theoretical decsription of the electron transport phenomena in several quantized systems, such as quantum wells, quantum wires, and superlattices (SLs), has been given in earlier studies, and it is mainly based on the solution of the Boltzmann equation [4-6]. The use of SLs is important since increasing the dispersion relation of the minibands for carriers is possible [7]. Therefore, this means that different origins of the periodic electron/hole potential, which take place in the compositional SLs and in the SLs formed by selective doping, can cause different consequences, influencing the formation of the miniband structures, altering the electrical conductivity, and affecting the electron scattering [6]. However, most of those studies treat only *n*-type systems, and very little has been reported in the literature regarding *p*-type materials, including experimental results [8-10].

In this study, the behavior of the electrical conductivity in *p*-type GaAs/Al_0.3_Ga_0.7_As and cubic GaN/Al_0.3_Ga_0.7_N SLs with thin barrier and well layers is studied. A self-consistent $\vec{k}\cdot \vec{p}$ method [11-13] is applied, in the framework of the effective-mass theory, which solves the full 6 × 6 Luttinger-Kohn (LK) Hamiltonian, in conjunction with the Poisson equation in a plane wave representation, including exchange-correlation effects within the local density approximation (LDA). The calculations were carried out at zero temperature and low-field limits, and the collision integral was taken within the framework of the relaxation time (τ) approximation.

The III-N semiconductors present both phases: the stable wurtzite (*w*) phase, and the cubic (*c*) phase. Although most of the progress achieved so far is based on the wurtzite materials, the metastable *c*-phase layers are promising alternatives for similar applications [14,15]. Controlled *p*-type doping of the III-N material layers is of crucial importance for optimizing electronic properties as well as for transport-based device performance. Nevertheless, this has proved to be difficult by virtue of the deep nature of the acceptors in the nitrides (around 0.1-0.2 eV above the top of the valence band in the bulk materials), in contrast with the case of GaAs-derived heterostructures, in which acceptor levels are only few meV apart from the band edge [9,11]. One way to enhance the acceptor doping efficiency, for example, is the use of SLs which create a two-dimensional hole gas (2DHG) in the well regions of the heterostructures. Contrary to the case of wurtzite material systems, in *p*-doped cubic structures, a 2DHG may arise, even in the absence of piezoelectric (PZ) fields [16]. The emergence of the 2DHG, is the main reason for the realization of our calculations in cubic phase; the PZ fields can decrease drastically the dispersion relation and consequently the conductivity [17,18].

The results obtained in this study constitute the first attempt to calculate electron conductivity in *p*-type SLs in the direction perpendicular to the layers and will be able to clarify several aspects related to transport properties.

## Theoretical model

The calculations were carried out by solving the 6 × 6 LK multiband effective mass equation (EME), which is represented with respect to a basis set of plane waves [11-13]. One assumes an infinite SL of squared wells along <001> direction. The multiband EME is represented with respect to plane waves with wavevectors *K *= (2π/*d*)*l *(*l *integer, and *d *the SL period) equal to reciprocal SL vectors. Rows and columns of the 6 × 6 LK Hamiltonian refer to the Bloch-type eigenfunctions $|jm_{j}^{}\vec{k}\rangle$ of Γ_8 _heavy and light hole bands, and Γ_7 _spin-orbit-split-hole band; $\vec{k}$ denotes a vector of the first SL Brillouin zone.

Expanding the EME with respect to plane waves 〈*z*|*K*〉 means representing this equation with respect to Bloch functions $\langle \vec{r}|m_{j}\vec{k}+K{\hat{e}}_{z}\rangle$. For a Bloch-type eigenfunction $\langle z|E\vec{k}\rangle$ of the SL of energy *E *and wavevector $\vec{k}$, the EME takes the form:

(1) $$\sum_{j^{\prime }{m^{\prime }}_{j}K}\langle jm_{j}\vec{k}K|T+V_{\text{A}}+V_{\text{H}}+V_{\text{HET}}+V_{\text{XC}}|j^{\prime }{m^{\prime }}_{j}\vec{k}K\rangle \langle j^{\prime }{m^{\prime }}_{j}\vec{k}K^{\prime }|v\vec{k}\rangle =E_{v}(\vec{k})\langle jm_{j}\vec{k}K|v\vec{k}\rangle$$

where *T *is the effective kinetic energy operator including strain, *V*_HET _is the valence and conduction band discontinuity potential, which is diagonal with respect to *jm_j _*, $j'm_{j}^{'}$, *V*_A _is the ionized acceptor charge distribution potential, *V*_H _is the Hartree potential due to the hole- charge distribution, and *V*_XC _is the exchange-correlation potential considered within LDA. The Coulomb potential, given by contributions of *V*_A _and *V*_H_, is obtained by means of a self-consistent procedure, where the Poisson equation stands, in reciprocal space, as presented in detail in refs. [11,12].

According to the quasi-classical transport theory based on Boltzmann's equation with the collision integral taken within the relaxation time approximation, the conductivity for vertical transport in SL minibands at zero temperature and low-field limit can be written as

(2) $$\sigma_{q}(E_{\text{F}})=\sum_{v}e^{2}\frac{\tau_{q,v}}{\hbar^{2}}\frac{1}{4\pi^{2}}\int_{ZB}d^{3}k{\left(\frac{\partial E_{q,v}(k)}{\partial k_{z}}\right)}^{2}\delta \left(E_{\text{F}}-E_{q,v}(k)\right),\;q=\text{hh,hl,so}$$

where the relaxation time *τ_qv _*is ascribed to the band *E_q,v _*, and hh, lh, and so, respectively, denote heavy hole, light hole and split-off hole. Introducing *σ_q_*(*E*_F_) as the conductivity contribution of band *E_q,v _*, one can write

(3) $$\sigma_{q}(E_{\text{F}})=\sum_{v}\sigma_{q,v}(E_{\text{F}})$$

(4) $$\sigma_{q,v}(E_{\text{F}})=\frac{e^{2}\tau_{q,v}}{m_{q}{}^{*}}\eta_{q,v}^{\text{eff}}(E_{\text{F}})$$

where

(5) $$\eta_{q,v}^{\text{eff}}(E_{\text{F}})=\frac{1}{2\pi^{2}}{\left(\frac{m_{q}{}^{*}}{\hbar^{2}}\right)}^{2}\int_{BZ}dk_{z}\theta (E_{\text{F}}-\varepsilon_{q,v}(k_{z})){\left(\varepsilon_{q,v}(k_{z})\right)}^{2}$$

**Figure 1:**
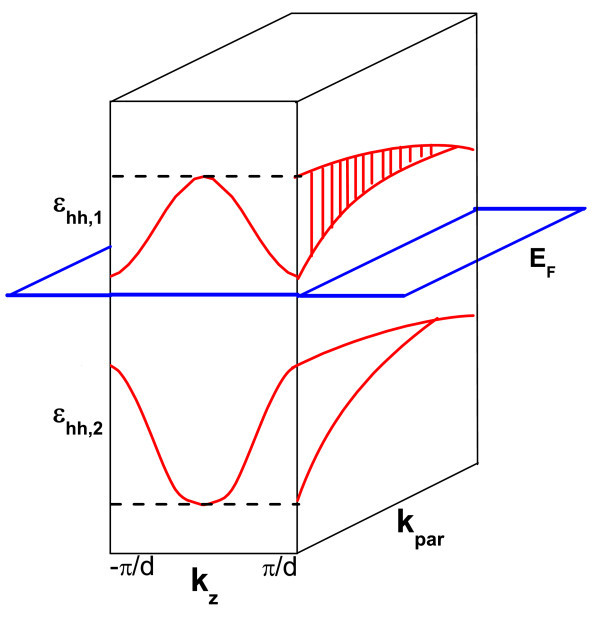
**Schematic representation of a SL band structure used in this study**. Minibands for heavy hole levels, *ε*_hh,1 _, minigaps, subbands, and Fermi level, *E*_F_, are shown. The zero of energy was considered at the top of the Coulomb potential at the barrier. Horizontal dashed lines indicate the bottom of the first miniband and the top of the second miniband, respectively.

The prime indicates the derivative of *ε_q,v_*(*k_z_*) with respect to *k_z _*. Once the SL miniband structure is accessed, *σ_q _*can be calculated, provided that the values of *τ_q,v _*are known. The relaxation time for all the minibands is assumed to be the same. In order to describe qualitatively the origin of the peculiar behavior as a function of *E*_F _, Equation (5) is analyzed with the aid of the SL band structure scheme as shown in Figure 1. It is important to see that minibands are presented just for heavy hole levels, since only they are occupied. Let us assume that *E*_F _moves down through the minibands and minigaps as shown in the figure. One considers the zero in the top of the Coulomb barrier. The density $n_{q,\nu }^{\text{eff}}(E_{\text{F}})$ is zero if *E*_F _lies up at the maximum (Max) of a particular miniband *ε_q,v _*. Its value rises continuously as *E*_F _spans the interval between the bottom and the top of this miniband. For *E*_F _smaller than the minimum (Min) of this miniband, $n_{q,\nu }^{\text{eff}}(E_{\text{F}})$ remains constant. A straightforward analysis of Equation (5) shows that *σ_q _*increases as *E*_F _crosses a miniband and stays constant as *E*_F _crosses a minigap. Therefore, a plateau-like behavior is expected for *σ_q _*as a function of *E*_F_. For a particular SL of period *d*, one moves the Fermi level position down through a minigap by increasing the acceptor-donor concentration *N*_A_, so the same behavior is expected for *σ_q _*as a function of *N*_A_. This fact was reported previously for *n*-type delta doping SLs [4].

In this way, we have the following expression for $n_{q,\nu }^{\text{eff}}(E_{\text{F}})$:

(6) $$n_{q,\nu }^{\text{eff}}(E_{\text{F}})=\frac{1}{2\pi^{2}}{\left(\frac{m_{q}{}^{*}}{\hbar^{2}}\right)}^{2}\cdot \left\{ \begin{array}{l} \;0\;E_{\text{F}}\langle \text{ Max }(\varepsilon_{q,\nu })\\ \int_{-k_{F\nu }}^{k_{F\nu }}dk_{z}{\left[{\varepsilon^{\prime }}_{q,\nu }^{}\right]}^{2}\text{ Max}(\varepsilon_{q,\nu })\;\langle \;E_{\text{F}}\;\langle \text{ Min}(\varepsilon_{q,\nu })\\ \int_{-\pi /d}^{\pi /d}dk_{z}{\left[{\varepsilon^{\prime }}_{q,\nu }^{}\right]}^{2}\;E_{\text{F}}\;\rangle \text{ Min}(\varepsilon_{q,\nu }) \end{array}\right.$$

The parameters used in these calculations are the same as those used in our previous studies [11-13]. In the above calculations, 40% for the valence-band offset and relaxation time τ = 3 ps has been adopted [19].

## Results and discussion

**Figure 2:**
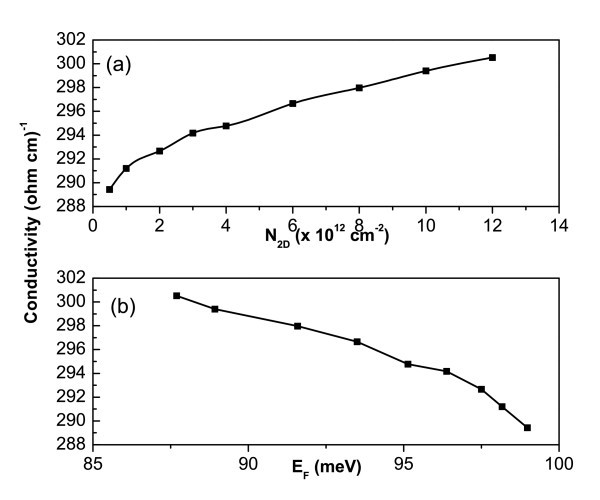
**Conductivity behavior for vertical transport in *p*-type GaAs/Al_0.3_Ga_0.7_As SLs with barrier and well widths equal to 2 nm, as a function of (a) the acceptor concentration *N*_2D _and (b) the Fermi energy *E*_F_**.

Figure 2a shows the conductivity for heavy holes (σ as a function of the two-dimensional acceptor concentration, *N*_2D_, for unstrained GaAs/Al_0.3_Ga_0.7_As SLs with barrier width, *d*_1 _= 2 nm, and well width, *d*_2 _= 2 nm). The conductivity increases until *N*_2D _= 3 × 10^12 ^cm^-2 ^because of the upward displacement of the Fermi level, which moves until the first miniband is fully occupied. Afterward, one observes a small range of concentrations with a plateau-like behavior for the conductivity; this is a region where there is no contribution from the first miniband or where the second band is partially occupied, but its contribution to the conductivity is very small. In the group-III arsenides, the minigap is shorter due to the lower values of the effective masses. After *N*_A _= 4 × 10^12 ^cm^-2, ^the conductivity increases again because of occupation of the second miniband, and this being very significant in this case. Figure 2b indicates the Fermi level behavior as a function of *N*_2D_, where the zero of energy is adopted at the top of the Coulomb barrier, as mentioned before. It is observed that the Fermi energy decreases as *N*_2D _increases. This happens because of the exchange-correlation effects, which play an important role in these structures. These effects are responsible for changes in the bending of the potential profiles. The bending is repulsive particularly for this case of GaAs/AlGaAs, and so the Coulomb potential stands out in relation to the exchange-correlation potential.

**Figure 3:**
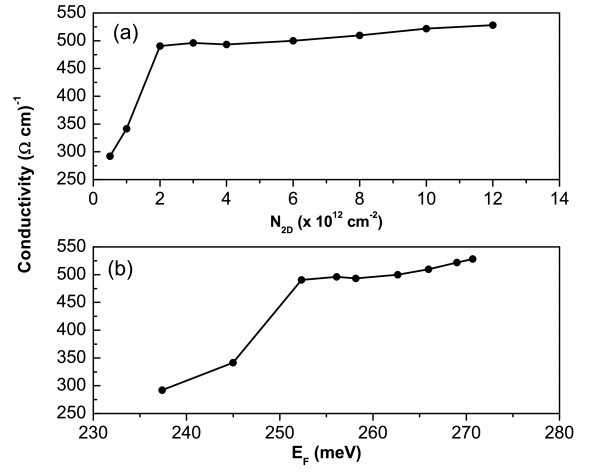
**Conductivity behavior for vertical transport in *p*-type GaN/Al_0.3_Ga_0.7_N SLs with barrier and well widths equal to 2 nm, as a function of (a) the acceptor concentration *N*_2D _and (b) the Fermi energy *E*_F_**.

Figure 3a depicts the conductivity behavior of heavy holes as a function of *N*_2D _for unstrained GaN/Al_0.3_Ga_0.7_N SLs with barrier width, *d*_1 _= 2 nm, and well width *d*_2 _= 2 nm. In this case, the conductivity increases until *N*_2D _= 2 × 10^12 ^cm^-2 ^and afterward it remains constant, until *N*_2D _= 6 × 10^12 ^cm^-2^. A simple joint analysis of Figure 3a,b can provide the correct understanding of this behavior. At the beginning, the first miniband is only partially occupied; once the band filling increases, i.e., as the Fermi level goes up to the first miniband value, the conductivity increases. When the occupation is complete (*N*_2D _= 2 × 10^12 ^cm^-2^), one reaches a plateau in the conductivity. After the second miniband begins to get filled up, σ is found to increase again. However, it is important to note that, for the nitrides, the Fermi level shows a remarkable increase as *N*_2D _increases, a behavior completely different as compared to that of the arsenides. This can be explained in the following way: for thinner layers of nitrides, the exchange-correlation potential effects are stronger than the Coulomb effects, and so the potential profile is attractive, and it is expected that the Fermi level goes toward the top of the valence band, as well as the miniband energies. This has been discussed in our previous study describing a detailed investigation about the exchange-correlation effects in group III-nitrides with short period layers [13].

Comparing both the systems (Figures 2 and 3), one can observe higher conductivity values for the nitride; several factors can contribute to this behavior, such as the many body effects as well as the values of effective masses, involved in the calculations of the densities $n_{q,\nu }^{\text{eff}}(E_{\text{F}})$. Experimental results for *p*-doped cubic GaN films, which use the concept of reactive co-doping, have obtained vertical conductivities as high as 50/Ωcm [8]. Those results corroborate with those of this study, since in the case of SLs, higher values for the conductivity are expected. Another interesting point concerning the arsenides relates to the higher values found for their conductivity in the case of systems, e.g., *n*-type delta doping GaAs system. The reason is the same as that given earlier.

## Conclusions

In conclusion, this investigation shows that the conductivity behavior for heavy holes as a function of *N*_2D _or of the Fermi level depicts a plateau-like behavior due to fully occupied levels. A remarkable point refers to the relative importance of the Coulomb and exchange-correlation effects in the total potential profile and, consequently, in the determination of the conductivity. These results presented here are expected to be treated as a guide for vertical transport measurements in actual SLs. Experiments carried out with good quality samples, combined with the theoretical predictions made in this study, will provide the way to elucidate the several physical aspects involved in the fundamental problem of the conductivity in SLs minibands.

## Abbreviations

2DHG: two-dimensional hole gas; EME: effective mass equation; LDA: local density approximation; PZ: piezoelectric; SLs: superlattices.

## Competing interests

The authors declare that they have no competing interests.

## Authors' contributions

OFPS carried out the calculations. GMS, LMRS and EFSJ discussed the results and purposed new calculations and improvements. SCPR conceived of the study and participated in its design and coordination. All authors read and approved the final manuscript.
